# Risk prediction nomogram for major morbidity related to primary resection for esophageal squamous cancer

**DOI:** 10.1097/MD.0000000000026189

**Published:** 2021-08-06

**Authors:** Xiao-long Liu, Rong-chun Wang, Yi-yang Liu, Hao Chen, Chen Qi, Li-wen Hu, Jun Yi, Wei Wang

**Affiliations:** a Department of Cardiothoracic Surgery, Jingling Hospital, Jingling School of Clinical Medicine, Nanjing Medical University; b Department of Cardiothoracic Surgery, Jingling Hospital, Medical School of Nanjing University; c Department of Thoracic Surgery, the First Affiliated Hospital of Nanjing Medical University, Nanjing, China.

**Keywords:** esophageal squamous cancer, major morbidity, nomogram, risk prediction

## Abstract

**Background and Objectives:**

Postoperative major complications after esophageal cancer resection vary and may significantly impact long-term outcomes. This study aimed to build an individualized nomogram to predict post-esophagectomy major morbidity.

**Methods:**

This retrospective study included 599 consecutive patients treated at a single center between January 2017 and April 2019. Of them, 420 and 179 were assigned to the model development and validation cohorts, respectively. Major morbidity predictors were identified using multiple logistic regression. Model discrimination and calibration were evaluated by validation. Regarding clinical usefulness, we examined the net benefit using decision curve analysis.

**Results:**

The mean age was 64 years; 79% of the patients were male. The most common comorbidities were hypertension, diabetes mellitus, and stroke history. The 30-day postoperative major morbidity rate was 24%. Multivariate logistic regression analysis showed that age, smoking history, coronary heart disease, dysphagia, body mass index, operation time, and tumor size were independent risk factors for surgery-associated major morbidity. Areas under the receiver-operating characteristic curves of the development and validation groups were 0.775 (95% confidence interval, 0.721–0.829) and 0.792 (95% confidence interval, 0.709–0.874), respectively. In the validation cohort, the nomogram showed good calibration. Decision curve analysis demonstrated that the prediction nomogram was clinically useful.

**Conclusion:**

Morbidity models and nomograms incorporating clinical and surgical data can be used to predict operative risk for esophagectomy and provide appropriate resources for the postoperative management of high-risk patients.

## 1 Introduction

Although treatment paradigms for esophageal cancer have changed significantly over the past decade, esophagectomy remains the mainstay treatment for most patients with esophageal cancer selected to undergo curative treatment.^[1–4]^ However, the associated postoperative mortality and morbidity rates are 1% to 6% and 19% to 60%, respectively, although outcomes at experienced high-volume centers tend to be better.^[5–7]^ Complications can range from minor complications (atelectasis) to severe complications (sepsis). Some studies have confirmed that anastomotic leakage and other related complications in cervical anastomosis are relatively high after esophagectomy. Such high complications occur because of surgery involving a wide range of areas including the abdomen, chest, and neck as well as decreased oxygen supply and increased tension from the gastric tube. However, some surgeons prefer neck anastomosis because it involves a lower rate of serious postoperative complications than intrathoracic anastomosis.^[8,9]^

Most studies that aimed to improve esophageal cancer surgery outcomes focused on long-term survival as the main outcome. However, postoperative major morbidity is an undesirable but critical outcome for both clinicians and patients with esophageal cancer. If adjuvant treatment is required, the timely initiation of chemotherapy or radiotherapy is delayed, which tends to impair overall survival.^[10,11]^ This might be related to postoperative weight loss and worsened nutritional status, which could, along with preexisting comorbidities, lead to a performance status decline and worse overall survival rate.^[12]^ Thus, it is important to identify subgroups that are at a higher risk of major morbidity after esophageal cancer surgery.

Some studies have suggested prediction models using the identified risk factors to predict the occurrence of postoperative complications in general, cardiac, and hepatocellular cancer surgery.^[13–16]^ Several studies have used nomograms to predict mortality and morbidity; however, they focused on clinical factors for complications after esophagectomy, which did not result in reliable risk models^[17,18]^ except for the prediction of pulmonary complications.^[19]^ Other previous predictions focused only on preoperative indicators and mortality for complications after esophagectomy, and their external validation results were unreliable.^[20]^ Models that focus on the presence of unspecified complications cannot be used in esophageal surgery owing to the large variation in complications. Owing to the lack of an effective tool for estimating the individual risk of postoperative major morbidity in the field of esophageal cancer, we aimed to build and evaluate a morbidity risk prediction model for a population of Chinese patients with esophageal cancer treated with surgical resection.

## 2 Materials and methods

### 2.1 Patient selection

**Figure 1:**
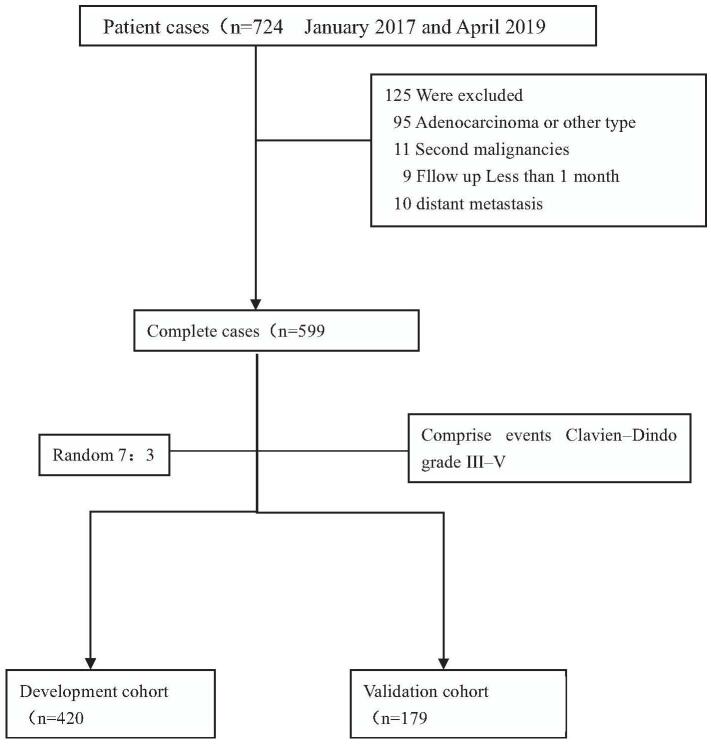
Flowchart depicting consecutive patients and reasons for exclusion from the present study.

This retrospective cohort study was performed in a single large and comprehensive medical center at Jin Ling Hospital, Nanjing, China. Data were obtained from the database of consecutive patients with esophageal squamous cancer treated between January 2017 and April 2019. All patients underwent a standard diagnostic workup including endoscopy with histological biopsy, endoscopic ultrasonography, computed tomography of the chest and abdomen, and external ultrasonography of the neck. Positron emission tomography was not routinely performed during the study period. However, it was optionally used to rule out cases of suspected metastasis, and adjuvant therapy was chosen as needed. All other pathologic cases of adenocarcinoma or other cell types or with distant metastasis, secondary malignancies, or a follow-up period <1 month were excluded from the study (Fig. 1). The Institutional Ethics Committee approved the study and the procedures followed were in accordance with the principles of the Declaration of Helsinki.

### 2.2 Variables

Clinical variables selected for the analysis included age at the time of surgery, sex, presence of comorbidities (diabetes mellitus [DM], hypertension, chronic obstructive pulmonary disease [COPD], coronary heart disease [CHD], stroke history), dysphagia symptoms, body mass index (BMI), drinking and smoking history of >20 years, and American Society of Anesthesiologists (ASA) score.^[21]^ Surgical data included operation time, number of lymph node dissections, anastomosis position (cervical or intrathoracic), type of surgery (open operation, conventional minimally invasive esophagectomy [MIE], and robot-assisted MIE), tumor length, and tumor location (upper, middle, lower). The other variables added were T- stage and N-stage from the American Joint Committee on Cancer 8th edition cancer staging system. Neoadjuvant chemoradiotherapy has been recommended for cT3-T4 or cN+ patients since the end of the last decade. It was performed with a taxane- and platinum-based double regimen. Two preoperative cycles were routinely recommended. Radiation was delivered by three-dimensional conformal radiotherapy or intensity-modulated radiotherapy. The total radiation dose was at least 50 Gy delivered in conventional fractions. Meanwhile, preoperative laboratory values including white blood cell count and albumin and carcinoembryonic antigen levels were collected.

### 2.3 Definition of complications

The Clavien–Dindo modified classification^[22]^ of complications was used to define morbidity. Major morbidity was defined as an event greater or equal to grade III that occurred up to 30 days after surgery, both during the hospital stay or after discharge. The same 30-day period was used when mortality was reported.

### 2.4 Statistical analysis

Quantitative data are presented through adequate measures of central tendency and dispersion (mean and standard deviation or median and interquartile range). Categorical variables are presented as frequencies and proportions. Variable factor analysis was performed using univariate and multivariate logistic regression analyses. Variables showing statistical significance in the univariate analysis were included in the multivariate logistic regression analysis, and the forward stepwise method was used to select the variables that were eventually included in the model. Logistic regression models were built to identify independent predictors of morbidity. For model building, a group of seven variables was initially included into the model, including age, smoking history, BMI, dysphagia, CHD, operation time, and tumor size. The assumption of linearity was assessed for all continuous variables. No imputation method was used for the missing data. A nomogram was constructed with coefficients from the final models, and their attributable points are shown in Table 1. For the model performance assessment, its capacity to discriminate between events and nonevents was assessed through receiver-operating characteristic curves and the c-statistic, which represents the area under the curve (AUC). Calibration was performed to investigate the goodness of fit of the prediction model. Finally, to evaluate the performance of the prediction models, we introduced a newly developed analysis technique (decision curve analysis), which is based on the principle that the relative harm of false positives and false negatives can be expressed in terms of a probability threshold, where n is the total number of patients in the study and Pt is the given threshold probability. All statistical analyses were conducted using Empower(R) (www.empowerstats.com; X&Y Solutions, Inc., Boston, MA) and R software (version 3.4.3; www.r-project.org). The significance level was set at 0.05, and all tests were 2-sided.

**Table 1:** Univariate and multivariate logistic regression models in the development group.

	Univariate analysis OR (95% CI) *P*	Multivariate analysis OR (95% CI) *P*
Age	1.04 (1.01–1.07) .0077	1.05 (1.01–1.08) .0087
Smoking	2.57 (1.63–4.06) <.0001	2.45 (1.47–4.09) .0006
Dinking	1.37 (0.88–2.14) .1638	
Sex	1.75 (0.94–3.27) .0793	
DM	0.85 (0.36–2.04) .7236	
HTN	1.26 (0.79–2.02) .3348	
COPD	3.04 (0.75–12.37) .1207	
CHD	5.94 (2.14–16.50) .0006	4.94 (1.58–15.49) .0061
Stroke	1.27 (0.54–2.99) .5878	
Dysphagia	5.08 (2.64–9.76) <.0001	5.82 (2.81–12.03) <.0001
BMI	0.90 (0.84–0.97) .0038	0.90 (0.83–0.98) .0111
Operation time	1.50 (1.22–1.83) <.0001	1.48 (1.18–1.85) .0006
Tumor size	1.23 (1.08–1.41) .0023	1.23 (1.06–1.43) .0074
Location		
Upper	1.0	
Middle	2.25 (0.64–7.91) .2069	
Lower	1.54 (0.42–5.57) .5133	
ASA		
1	1.0	
2	0.57 (0.33–0.99) .0449	
3	2.12 (0.92–4.89) .0766	
4	0.54 (0.06–4.70) .5773	
Type of operation		
Open operation	1.0	
MIE	1.95 (1.20–3.18) .0072	
RAMIE	2.79 (1.42–5.49) .0029	
Anastomosis		
Cervical	1.0	
Intrathoracic	0.67 (0.40–1.11) .1225	
Lymph no.	1.02 (1.00–1.05) .0323	
Tstage		
TIS	1.0	
T1	0.87 (0.26–3.00) .8317	
T2	0.70 (0.21–2.39) .5689	
T3	1.30 (0.40–4.19) .6655	
T4	1.12 (0.20–6.43) .8947	
Nstage		
N0	1.0	
N1	1.16 (0.69–1.94) .5743	
N2	1.28 (0.66–2.49) .4700	
N3	2.43 (0.53–11.16) .2543	
NC	0.91 (0.47–1.76) .7693	

## 3 Results

### 3.1 Patient demographics

In this study, 599 consecutive patients were enrolled, including 420 in the development group and 179 in the validation group. There were 477 men and 122 women (median age, 65 years; range, 59–69 years). A total of 42 patients had DM, 189 had hypertension, and 240 had a history of smoking and drinking. A total of 13, 34, and 22 patients had COPD, stroke history, and CHD, respectively. More than half of the patients had a primary tumor located in the middle esophagus and an anastomosis site in the cervical region. The median tumor size was 3.0 (range, 2.0–4.5) cm, median operation time was 3.80 (range, 2.90–4.55) hours, median BMI was 23.00 (range, 21.00–25.00) kg/m^2^, and median number of harvested lymph nodes was 20.00 (range, 14.00–27.00) (Table 2). Most of the patients were staged as T2–3, with <50% having a node-positive disease. Neoadjuvant radiochemotherapy was administered to 13% of the patients. Comparison of the baseline data indicated no differences in the general characteristics of patients in the development and validation groups.

**Table 2:** Baseline characteristics of the development group and validation group (n = 599).

Group	Development (n = 420)	Validation (n = 179)	*P*
Age (median, Q1–Q3)	64.00 (59.00–69.00)	65.00 (60.00–70.00)	.264
BMI, kg/m^2^ (Median, Q1–Q3)	23.00 (21.00–25.00)	23.00 (21.00–25.00)	.746
Smoking history			.835
No	219 (52.14%)	95 (53.07%)	
Yes	201 (47.86%)	84 (46.93%)	
Dinking history			.659
No	250 (59.52%)	110 (61.45%)	
Yes	170 (40.48%)	69 (38.55%)	
Sex			.219
Female	80 (19.05%)	42 (23.46%)	
Male	340 (80.95%)	137 (76.54%)	
DM			.588
No	389 (92.62%)	168 (93.85%)	
Yes	31 (7.38%)	11 (6.15%)	
HTN			.289
No	293 (69.76%)	117 (65.36%)	
Yes	127 (30.24%)	62 (34.64%)	
COPD			.495
No	412 (98.10%)	174 (97.21%)	
Yes	8 (1.90%)	5 (2.79%)	
CHD			.938
No	403 (95.95%)	172 (96.09%)	
Yes	17 (4.05%)	7 (3.91%)	
Stroke history			.349
No	393 (93.57%)	171 (95.53%)	
Yes	27 (6.43%)	8 (4.47%)	
Dysphagia			.188
No	377 (89.76%)	154 (86.03%)	
Yes	43 (10.24%)	25 (13.97%)	
Tumor location			.224
Upper	20 (4.76%)	15 (8.38%)	
Middle	250 (59.52%)	103 (57.54%)	
Lower	150 (35.71%)	61 (34.08%)	
ASA			.932
1	274 (65.24%)	121 (67.60%)	
2	115 (27.38%)	45 (25.14%)	
3	25 (5.95%)	11 (6.15%)	
4	6 (1.43%)	2 (1.12%)	
Operation time, h	3.90 (2.90–4.60)	3.60 (2.80–4.50)	.761
Tumor size, cm	3.50 (2.30–4.50)	3.00 (2.20–4.35)	.720
Lymph no.	20.00 (14.00–26.00)	21.00 (15.00–27.50)	.351
Type of operation			.278
Open operation	200 (47.62%)	81 (45.25%)	
MIE	170 (40.48%)	68 (37.99%)	
RAMIE	50 (11.90%)	30 (16.76%)	
Anastomosis			.269
Cervical	296 (70.48%)	118 (65.92%)	
Intrathoracic	124 (29.52%)	61 (34.08%)	
Tstage			.623
TIS	16 (3.81%)	8 (4.47%)	
T1	93 (22.14%)	33 (18.44%)	
T2	111 (26.43%)	50 (27.93%)	
T3	189 (45.00%)	86 (48.04%)	
T4	11 (2.62%)	2 (1.12%)	
Nstage			.936
N0	250 (59.52%)	104 (58.10%)	
N1	110 (26.19%)	49 (27.37%)	
N2	53 (12.62%)	24 (13.41%)	
N3	7 (1.67%)	2 (1.12%)	
NC			.935
No	365 (86.90%)	156 (87.15%)	
Yes	55 (13.10%)	23 (12.85%)	

### 3.2 Morbidity outcome

A total of 218 patients had complications. The overall 30-day morbidity rate was 36.3%. The most common morbidities were pneumonia and wound site infection. The number of Clavien I (atelectasis, vocal cord paresis or paralysis) was 41(6.8%); the number of Clavien II (pneumonia, chyle leakage, pulmonary embolus) was 29 (4.8%); major morbidity occurred in 24.6% of subjects and half of the events were classified as Clavien III. The number of Clavien III was 72 (12%), the Anastomotic leak number was 48 (8%), reoperation due to thoracic duct injury was 14 (2.3%); the number of Clavien IV was 50 (8.3%), requiring artificial ventilation was 28 (4.8%), heart failure was 8 (1.3%), renal insufficiency was 3 (0.5%), combination of at least 2 complications was 11 (1.8%); the number of Clavien V (30- day postoperative death) was 26 (4.3%) (Table 3).

**Table 3:** Overall and major complications after esphogectomy.

Complication category	Complications	No.	%
Overall morbidity	I–V	218	36.3
	Clavien I		
	Atelectasis, vocal cord paresis or paralysisClavien II	41	6.8
	Pneumonia, chyle leakage, pulmonary embolus	29	4.8
Major morbidity	III–V	148	24.6
	Clavien III	72	12
	Anastomotic leak	48	8
	Reoperation due to thoracic duct injury	14	2.3
	Clavien IV	50	8.3
	Requiring artificial ventilation	28	4.8
	Heart failure	8	1.3
	Renal insufficiency	3	0.5
	Combination of at least 2 complications	11	1.8
	Clavien V (30-day postoperative death)	26	4.3

### 3.3 Nomogram development

**Figure 2:**
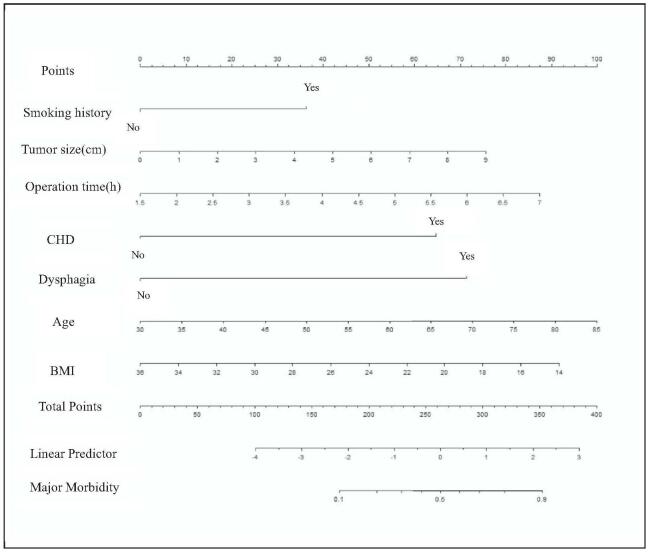
Nomogram predicting the probability of morbidity in patients after esophagectomy.

Univariate analysis of the development group showed that the statistically significant risk factors were age, smoking history, CHD, BMI, ASA, dysphagia, type of operation, operation time, tumor size, and number of lymph nodes (*P* < 0.05). Statistically significant variables on univariate analysis were included in the multivariate logistic regression analysis. Age, smoking history, CHD, BMI, operation time, tumor size, and dysphagia were independent risk factors for esophagectomy with morbidity (Table 2) (*P* < 0.05). Based on the logistic multivariate regression analysis, 7 independent risk factors were included in the prediction model. We then established an individualized nomogram prediction model of surgery-associated major morbidity (Fig. 2). Based on the nomogram, we can obtain the points corresponding to each prediction indicator. The sum of the points is recorded as the total score, and the predicted risk corresponding to the total score is the probability of surgery-associated morbidity.

### 3.4 Nomogram validation

**Figure 3:**
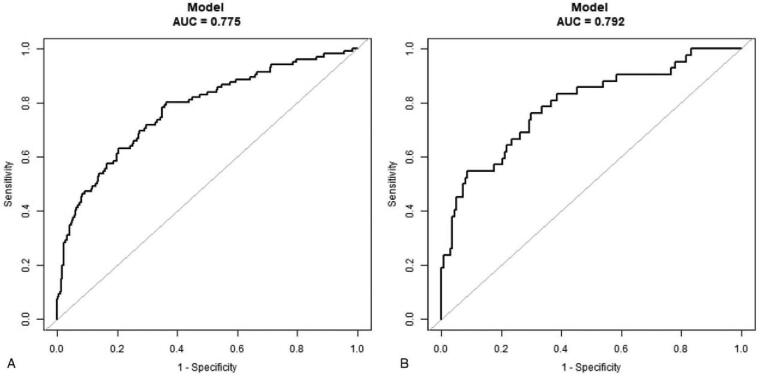
Receiver-operating characteristic curves for validating the discrimination power of the nomogram. Development group (A) and validation group (B) (area under the curve = 0.775 vs 0.792).

**Figure 4:**
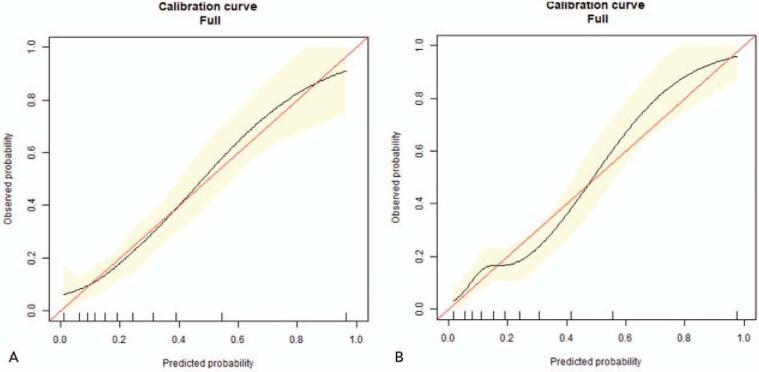
Calibration plots of the nomogram for the probability of morbidity in the development group (A) and validation group (B).

The validation of the model was based on discrimination and calibration. We drew the receiver-operating characteristic curves of predicted probability and calculated the AUC values for the development and validation groups. The AUC values for morbidity risk in the development and validation groups were 0.775 (95% confidence interval [CI], 0.721–0.829) and 0.792 (95% CI, 0.709–0.874) (Fig. 3A and B), respectively. The 95% CI of the calibration belt for the development and validation groups crossed the diagonal bisector line (Fig. 4A and B). Therefore, the predicted probability of the model was consistent with the actual probability, which suggested that the prediction model had strong concordant performance and the calibration of the prediction model in both groups was perfect.

### 3.5 Decision curve analysis

**Figure 5:**
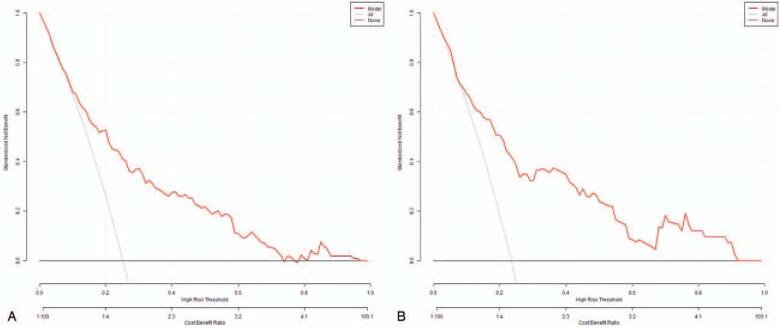
Decision curve analysis in the development group (A) and validation group (B).

Figure 5A and B illustrates the decision curves for models to predict the diagnosis of risk factors in patients after esophagectomy. The model was useful for threshold probabilities, and the net benefit of the validation group was better than that of the development group between the threshold probabilities.

## 4 Discussion

In the present study, a nomogram for predicting major morbidity after esophagectomy was constructed and validated internally. The factors incorporated in the model were age, smoking history, CHD, BMI, tumor size, dysphagia, and operation time. In terms of model performance, the constructed model showed good discrimination and calibration.

Postoperative complication rates are associated with a high morbidity rate of 19% to 60%.^[5]^ Nonetheless, the results have improved over time, and the postoperative mortality rate was <5% in the Japanese database.^[6,7]^ Esophagectomy procedures are usually complex and extensive; therefore, the postoperative morbidity rate is high. Models that focus on the presence of unspecified complications cannot be used in esophageal surgery because of the large variation in complications.^[23]^ The 30-day postoperative major morbidity rate in our study was 24%, which is similar to the previously reported rate. Postoperative morbidity adversely affects patients with esophageal cancer for several reasons, including escalating health care costs and increasing stress for patients and their families. Furthermore, if adjuvant treatment is required, the timely initiation of chemotherapy or radiotherapy is delayed, which is associated with impaired overall survival. Thus, identifying patients at a potential risk of postoperative morbidity is critical.

Several prediction models for postoperative morbidity have been introduced in general surgery, cardiac surgery, gastric cancer, and hepatocellular carcinoma populations.^[13]^ However, the aforementioned models could not be reliably used in esophageal cancer, and the predisposing risk factors for postoperative morbidity after esophagectomy for cancer did not result in reliable predictive models.^[24]^ Lagarde et al developed a nomogram to predict the occurrence and severity of complications in patients undergoing esophagectomy.^[25]^ The nomogram provides a graphical representation of the predictive strength of specific predictors and enables clinicians to calculate an overall risk score for individual patients that reflects their personal risk. Although Grotenhuis et al validated the nomogram, the validation population was closely related to the derivation population, indicating the need for further validation in a different surgical population. Meanwhile, the duration of enrolled consecutive series ultimately exceeded >10 years. However, the duration of the present study was only 2 years and it included only patients with pathologically proven esophageal squamous cell carcinoma. An interesting feature of our constructed nomogram is the incorporation of dysphagia. In the previously suggested models for morbidity and mortality, dysphagia was not analyzed as a potential predictor of postoperative morbidity. Patients with esophageal cancer frequently experience dysphagia to a degree that a reduction in food intake is apparent at the initial diagnosis. Malnutrition is associated with an increased risk of postoperative complications in patients undergoing esophagectomy.^[26–28]^

In the present study, ASA scores and type of operation, which were reported as predictors of postoperative morbidity in other studies, did not show a significant prognostic value. COPD was not a significant risk factor; however, smoking history was a risk factor. This finding may be explained by the fact that COPD diagnostic criteria are relatively strict considering a low ratio (<0.70) of the forced expiratory volume in 1 second. Therefore, the diagnostic COPD number was lower, and some smokers did not have airflow obstruction. In a previous longitudinal study, Woodruff et al found that respiratory symptoms were common in present or former smokers despite forced expiratory volume in 1 second: forced vital capacity and forced vital capacity values being generally normal.^[29]^ A previous study selected smoking history as a risk factor, which could adequately cover the complications associated with lung disease.^[30]^ This is consistent with a study reporting that smoking is a risk factor for postoperative morbidity, whereas preoperative smoking cessation for >90 days is ideal to reduce morbidities after esophagectomy.^[31]^

Other studies reported that preoperative prediction of complications in individual patients remains difficult, most likely because of the complexity of the mechanisms causing these complications. However, these studies did not include operation time, tumor size, and cancer stage as risk factors. Our study findings are consistent with a study that demonstrated that MIE, minimally invasive Ivor Lewis and transhiatal esophagectomy in particular, is safe and equivalent to the open esophagectomy technique with respect to overall morbidity and mortality.^[32]^ Despite improvements in postoperative cancer surgery care, operative time remains an important and often overlooked predictor of complications. It was recently reported to be associated with morbidity after transhiatal and Ivor Lewis esophagectomies^[33]^ and an independent predictor of major morbidity in a Korean series along with pulmonary and cardiovascular comorbidities.^[34]^ The impact of operative time is likely to be experienced mostly among patients with worse performance status and in low-volume institutions, where mortality rates are historically higher.^[5]^ Our study demonstrated that operation time is correlated with major morbidity.

Our study presented and validated a user-friendly nomogram that generates a simple graphical quantification of postoperative morbidity probability. It incorporates both preoperative clinical parameters and surgical data to enhance the prediction of surgical outcomes in clinical practice and provide objective parameters for selecting specific populations, which is in line with the current trend toward personalized medicine and alternative treatment approaches. The quality of surgical care can be audited among both physicians and patients by comparing the expected rate of postoperative morbidity with the actual rate. In addition, to develop a reliable model, all clinical predictors should be tested for inclusion. The number of enrolled patients in the present study was sufficiently large to test candidate predictors. We performed decision curve analysis to assess the performance of the diagnostic models. The model was useful between threshold probabilities, and the net benefit of the validation group was better than that of the development group.

Although we achieved good results, the present study had some limitations. First, this retrospective study may have underestimated the morbidity rate. Nonetheless, all included patients received postoperative care at our institution, and the data were extracted from the linked database in 2016 in our institution. Therefore, this point may minimize the ascertainment bias. Second, although we performed rigorous internal validation, the nomogram still requires external validation by other institutions to gain general acceptance. A nomogram for routine practical use should be developed in a general population rather than in a selected group of patients treated in specialized hospitals. The specific characteristics of the population (robot-assisted MIE, esophageal squamous cancer) would likely render the nomogram minimally valuable elsewhere. Third, surgeon-related factors, such as skill and experience, and gastric tube length were not analyzed in the present nomogram model.

In summary, predictive parameters for postoperative complications after primary resection for esophageal squamous cancer are age, smoking history, coronary heart disease, dysphagia, body mass index, operation time, and tumor size. The nomogram was constructed based on logistic regression model. The point value assigned to each factor was proportional to the odds ratio derived from the beta coefficients for each factor determined by the regression analysis. This nomogram incorporates seven variables. For each level of each prognostic variable, points were allocated according to the scale shown. The total score was determined by adding individual parameter points and was used to calculate the predicted probability of 30-day major morbidity. A total score of 262 was assigned a value of 0.5 and used to define the groups at high-risk of 30-day major morbidity after primary resection for esophageal squamous cancer. Patients at higher risk should be given nutritional support before surgery to increase their weight, shorten the operation time, strengthen monitoring after surgery, and promptly find problems and deal with them in time.

## 5 Conclusion

We developed and internally validated an individualized nomogram for predicting major morbidity after esophagectomy. Using this prediction model, we can accurately predict the risk of esophagectomy, which helps to improve the understanding of operative risk and screening of high-risk patients.

## Acknowledgments

The authors thank Dr. Bin Yang for providing the design suggestions and statistical guidance. The authors are grateful to all the participants involved in the present study for their enthusiasm and commitment.

## Author contributions

**Conceptualization:** xiaolong liu, Rong-chun Wang, Wei Wang.

**Data curation:** Rong-chun Wang, Yi-yang Liu, Chen Qi.

**Funding acquisition:** Chen Qi, Wei Wang.

**Investigation:** Hao Chen, Chen Qi, Wei Wang.

**Methodology:** xiaolong liu, Yi-yang Liu, Hao Chen, Chen Qi.

**Resources:** xiaolong liu, Yi-yang Liu, Hao Chen, Chen Qi.

**Software:** Chen Qi.

**Supervision:** Li-wen Hu.

**Validation:** Li-wen Hu.

**Visualization:** Li-wen Hu.

**Writing – original draft:** xiaolong liu.

**Writing – review & editing:** Jun Yi, Wei Wang.
